# *Mucisphaera calidilacus* gen. nov., sp. nov., a novel planctomycete of the class *Phycisphaerae* isolated in the shallow sea hydrothermal system of the Lipari Islands

**DOI:** 10.1007/s10482-021-01707-3

**Published:** 2022-01-20

**Authors:** Nicolai Kallscheuer, Christian Jogler, Stijn H. Peeters, Christian Boedeker, Mareike Jogler, Anja Heuer, Mike S. M. Jetten, Manfred Rohde, Sandra Wiegand

**Affiliations:** 1 Department of Microbiology, Radboud University, Nijmegen, The Netherlands; 2 Department of Microbial Interactions, Institute of Microbiology, Friedrich Schiller University, Jena, Germany; 3 Leibniz Institute DSMZ, Braunschweig, Germany; 4 Central Facility for Microscopy, Helmholtz Centre for Infection Research, Braunschweig, Germany; 5 Institute for Biological Interfaces 5, Karlsruhe Institute of Technology, Eggenstein-Leopoldshafen, Germany

**Keywords:** Mediterranean Sea, Panarea, Red biofilm, *Phycisphaeraceae*, Binary fission, *Algisphaera*

## Abstract

For extending the current collection of axenic cultures of planctomycetes, we describe in this study the isolation and characterisation of strain Pan265^T^ obtained from a red biofilm in the hydrothermal vent system close to the Lipari Islands in the Tyrrhenian Sea, north of Sicily, Italy. The strain forms light pink colonies on solid medium and grows as a viscous colloid in liquid culture, likely as the result of formation of a dense extracellular matrix observed during electron microscopy. Cells of the novel isolate are spherical, motile and divide by binary fission. Strain Pan265^T^ is mesophilic (temperature optimum 30–33 °C), neutrophilic (pH optimum 7.0–8.0), aerobic and heterotrophic. The strain has a genome size of 3.49 Mb and a DNA G + C content of 63.9%. Phylogenetically, the strain belongs to the family *Phycisphaeraceae*, order *Phycisphaerales*, class *Phycisphaerae*. Our polyphasic analysis supports the delineation of strain Pan265^T^ from the known genera in this family. Therefore, we conclude to assign strain Pan265^T^ to a novel species within a novel genus, for which we propose the name *Mucisphaera calidilacus* gen. nov., sp. nov. The novel species is the type species of the novel genus and is represented by strain Pan265^T^ (= DSM 100697^T^ = CECT 30425^T^) as type strain.

## Introduction

*Planctomycetes* is a bacterial phylum currently harbouring around 130 described species. According to the current taxonomy, the phylum is subdivided into the classes *Planctomycetia* (≈ 100 species), *Phycisphaerae* (≈ 7 species) and *Candidatus* Brocadiae (≈ 20 species, all with *Candidatus* status due to the lack of axenic cultures). The phylum *Planctomycetes*, along with *Chlamydiae, Verrucomicrobia* and other sister phyla, belongs to the PVC superphylum, which has medical, environmental and biotechnological relevance (Wagner and Horn 2006).

Planctomycetes are frequently found to be associated with phytoplankton or other aquatic phototrophs, *e.g.* seagrasses or macroalgae (Bengtsson et al. 2012; Bondoso et al. 2015, 2014, 2017; Lage and Bondoso 2014; Vollmers et al. 2017). In that sense, planctomycetes contribute directly or indirectly to global biogeochemical cycles (Wiegand et al. 2018; Strous et al. 1999; Peeters and van Niftrik 2019). The observation that planctomycetes can survive in large oligotrophic waterbodies, *e.g.* oceans, led to the assumption that they are able to obtain carbohydrates from various biotic surfaces present in such waterbodies and thus also play a role in the global carbon cycle. This is in line with the observation that planctomycetes can be abundant members in microbial communities on biotic surfaces representing potential sources of nutrients (Delmont et al. 2018). Strains of the phylum *Planctomycetes* can *e.g.* account for up to 70% of the microbial community on the macroalga *Ecklonia radiata* on the Australian shore and even for more than 80% on leaves of the seagrass *Posidonia oceanica* in the Mediterranean Sea (Kohn et al. 2020; Wiegand et al. 2018). Such values point towards a metabolism well adapted to the utilisation of complex carbon sources derived from phototrophs (Jeske et al. 2013; Lachnit et al. 2013), which is also reflected by high numbers of genes found in the genomes of planctomycetes that are related to the degradation of high molecular weight sugars from phototrophs, *e.g.* genes coding for polysaccharide lyases or sulfatases (Elcheninov et al. 2017; Wegner et al. 2013). While several bacteria secrete catabolic enzymes for polysaccharide degradation and take up the cleavage products (monomers or oligomers) (Dunne et al. 2012; Johansen 2016), planctomycetes are suggested to follow an alternative and more ‘selfish’ strategy. Planctomycetes were shown to have an enlarged periplasmic space and form pili originating from conspicuous crateriform structures (Boedeker et al. 2017). These morphological features are probably part of a specialised system for the internalisation of entire polysaccharide molecules, in which the pili act as ‘fishing rods’ by binding to the polysaccharide molecule and the periplasmic space serves as a compartment for the temporary storage and digestion of the polymer. Polysaccharide uptake by planctomycetes was demonstrated for the model substrate dextran (Boedeker et al. 2017), a strategy that avoids providing easily available carbon sources to bacterial competitors that occupy the same ecological niche, *e.g.* species of the *Roseobacter* group (Frank et al. 2014). Such a strategy  is particularly important when considering that  planctomycetes (at least under laboratory-scale conditions) grow considerably slower than many other heterotrophic bacterial competitors found in the same ecosystems. Thus, planctomycetes need to follow other strategies than low generation times to achieve a growth advantage and thereby avoid to be outcompeted. Such ‘survival’ strategies may also involve the observed resistance of planctomycetes to several natural antibiotics (Cayrou et al. 2010; Godinho et al. 2019) and the ability to produce secondary metabolites with biological activities (Graça et al. 2016; Jeske et al. 2016; Kallscheuer et al. 2019, 2020c).

Several of the observed traits of planctomycetes, *e.g.* low growth speed and resistance to antibiotics are probably related to the uncommon cell biology of these bacteria, including differences in the mode of cell division. While strains of the class *Planctomycetia* divide by budding, members of the classes *Phycisphaerae* and *Cand*. Brocadiae divide by binary fission (Wiegand et al. 2020). Independent of the mode of cell division, all three classes lack many of the canonical divisome proteins, including the otherwise universal FtsZ (Jogler et al. 2012; Pilhofer et al. 2008; Wiegand et al. 2020).

In the past, additional exceptional traits of planctomycetes were proposed, such as lack of peptidoglycan (König et al. 1984), a compartmentalized cell plan (Lindsay et al. 1997), a nucleus-like structure (Fuerst and Webb 1991) and endocytosis (Lonhienne et al. 2010), indicating that planctomycetes are beyond the bacterial cell plan (Fuerst and Sagulenko 2011).

However, this picture changed with the advent of novel microscopic techniques and genetic tools for planctomycetes (Jogler et al. 2011; Jogler and Jogler 2013; Rivas-Marin et al. 2016). Planctomycetes were found to have peptidoglycan (Jeske et al. 2015; van Teeseling et al. 2015) and the proposed cell compartments were reinterpreted as invaginations of the cytoplasmic membrane (Acehan et al. 2013; Boedeker et al. 2017; Santarella-Mellwig et al. 2013), except for class *Cand.* Brocadiae (Jogler 2014; Neumann et al. 2014). Taken together, with some exceptions, the cell envelope architecture was reinterpreted as similar to that of Gram-negative bacteria (Boedeker et al. 2017; Devos 2014a, 2014b).

Despite the reinterpretation of the cell plan, planctomycetes are still fascinating. In most planctomycetal genomes, between 40 and 55% of the overall number of putative protein-coding genes are of unknown function, pointing towards a lot of undiscovered biology in the phylum *Planctomycetes* (Bordin et al. 2018; Overmann et al. 2017). As a contribution to the current collection of axenic cultures of planctomycetes, here we describe strain Pan265^T^ as a novel member of the sparsely studied class *Phycisphaerae*. This class was introduced in 2009 (Fukunaga et al. 2009) and currently holds seven described species belonging to the families *Phycisphaeraceae*, *Anaerohalosphaeraceae*, *Sedimentisphaeraceae* or *Tepidisphaeraceae* (Fukunaga et al. 2009; Kovaleva et al. 2015; Pradel et al. 2020; Spring 2015). In addition to binary fission as the mode of cell division (in contrast to budding in the class *Planctomycetia*), relatively small genomes (< 4.3 Mb) can be indicative for members of the class *Phycisphaerae* when compared to strains of the class *Planctomycetia*, which have typical genome sizes of 4.5–12.4 Mb.

## Material and methods

### Isolation of the novel strain and cultivation experiments

Strain Pan265^T^ was isolated from a red biofilm obtained from the hydrothermal vent system close to the Lipari Islands in the Tyrrhenian Sea (exact location 38.5568 N 15.1097 E). The sampling setup was identical as for strain Pan44^T^ described in a previous study (Kallscheuer et al. 2020d). Subsequent cultivation experiments were performed in M1 medium with 4-(2-hydroxyethyl)-1-piperazineethanesulfonic acid (HEPES) as buffering agent and additionally supplemented with *N*-acetyl glucosamine (NAG) and artificial seawater (ASW). This medium, designated M1H NAG ASW, was prepared as previously described (Boersma et al. 2020). The 16S rRNA gene of strain Pan265^T^ was amplified by PCR and sequenced prior to a detailed strain characterisation (Rast et al. 2017). The novel isolate was regarded as a member of the phylum *Planctomycetes* after blastn of the 16S rRNA gene sequence and identification of the closest hits as planctomycetes.

### Determination of pH and temperature optimum

Cultivation experiments for determination of the pH optimum were performed in M1H NAG ASW medium. For maintenance of the pH during the cultivation time, 100 mM of the following buffers was used: 2-(*N*-morpholino)ethanesulfonic acid (MES) for pH 5.0–6.5, HEPES for pH 7.0 and 7.5, 3-(4-(2-Hydroxyethyl)piperazin-1-yl)propane-1-sulfonic acid (HEPPS) for pH 8.0 and *N*-cyclohexyl-2-aminoethanesulfonic acid (CHES) for pH 9.0–10.0. Cultivation experiments for determination of the pH optimum were performed at 28 °C. Cultivation experiments for determination of the temperature optimum were performed at temperatures ranging from 5–40 °C in standard M1H NAG ASW medium at pH 7.5. Measurement of the optical density at 600 nm (OD_600_) was not possible for the strain due to growth as viscous colloid. Instead, optimal conditions were determined by visual inspection of cultures in biological triplicates.

### Microscopy protocols

Phase contrast light microscopy (LM) and field emission scanning electron microscopy (SEM) were performed as previously described (Boersma et al. 2019).

### Genome information and analysis of genome-encoded features

The genome and 16S rRNA gene sequence of strain Pan265^T^ are available from GenBank under accession numbers CP036280 and MK559984, respectively. Genome sequencing of the strain is part of a previous study (Wiegand et al. 2020). Numbers of carbohydrate-active enzymes were obtained from the CAZy database (Lombard et al. 2014). Gene clusters potentially involved in the production of secondary metabolites were predicted using antiSMASH bacterial version 5.1.2 with default parameters (relaxed strictness) (Blin et al. 2019).

### Phylogenetic analysis

The 16S rRNA gene sequence-based phylogeny was computed for strain Pan265^T^, the type strains of all described planctomycetal species (assessed in May 2021) including all isolates published and described in the last two years (Dedysh et al. 2019a, 2019b; Kallscheuer et al. 2020a, 2020b, 2020e; Kohn et al. 2020, 2019; Peeters et al. 2020; Rivas-Marin et al. 2020a, 2020b; Vitorino et al. 2020; Waqqas et al. 2020). The 16S rRNA gene sequences were aligned with SINA (Pruesse et al. 2012) and the phylogenetic inference was calculated with RAxML (Stamatakis 2014) with a maximum likelihood approach with 1,000 bootstraps, nucleotide substitution model GTR, gamma distributed rate variation and estimation of proportion of invariable sites (GTRGAMMAI option). For the multi-locus sequence analysis (MLSA) the unique single-copy core genome of the analysed genomes was determined with proteinortho5 (Lechner et al. 2011) with the ‘selfblast’ option enabled, a coverage of 50% and an e-value of 1e-05. The protein sequences of the resulting orthologous groups were aligned using MUSCLE v.3.8.31 (Edgar 2004). After clipping, partially aligned *C*- and *N*-terminal regions and poorly aligned internal regions were filtered using Gblocks with default settings (Castresana 2000). The final alignment was concatenated and clustered using the maximum likelihood method implemented by RAxML (Stamatakis 2014) with the ‘rapid bootstrap’ method and 500 bootstrap replicates using the amino acid substitution model PROTGAMMAIWAG. Strains part of the PVC superphylum (outside of the phylum *Planctomycetes*), namely *Opitutus terrae* (AJ229235), *Kiritimatiella glycovorans* (NR_146840) and *Lentisphaera araneosa* (NR_027571) served as outgroup in the 16S rRNA sequence-based tree, while *Opitutus terrae* (CP001032.1) and *Lacunisphaera limnophila* (CP016094.1) served as outgroup in the MLSA-based tree. The average nucleotide identity (ANI) was calculated using OrthoANI (Lee et al. 2016). The average amino acid identity (AAI) was calculated using the aai.rb script of the enveomics collection (Rodriguez-R and Konstantinidis 2016) and the percentage of conserved proteins (POCP) was calculated as described (Qin et al. 2014).

## Results and discussion

### Phylogenetic inference

**Fig. 1:**
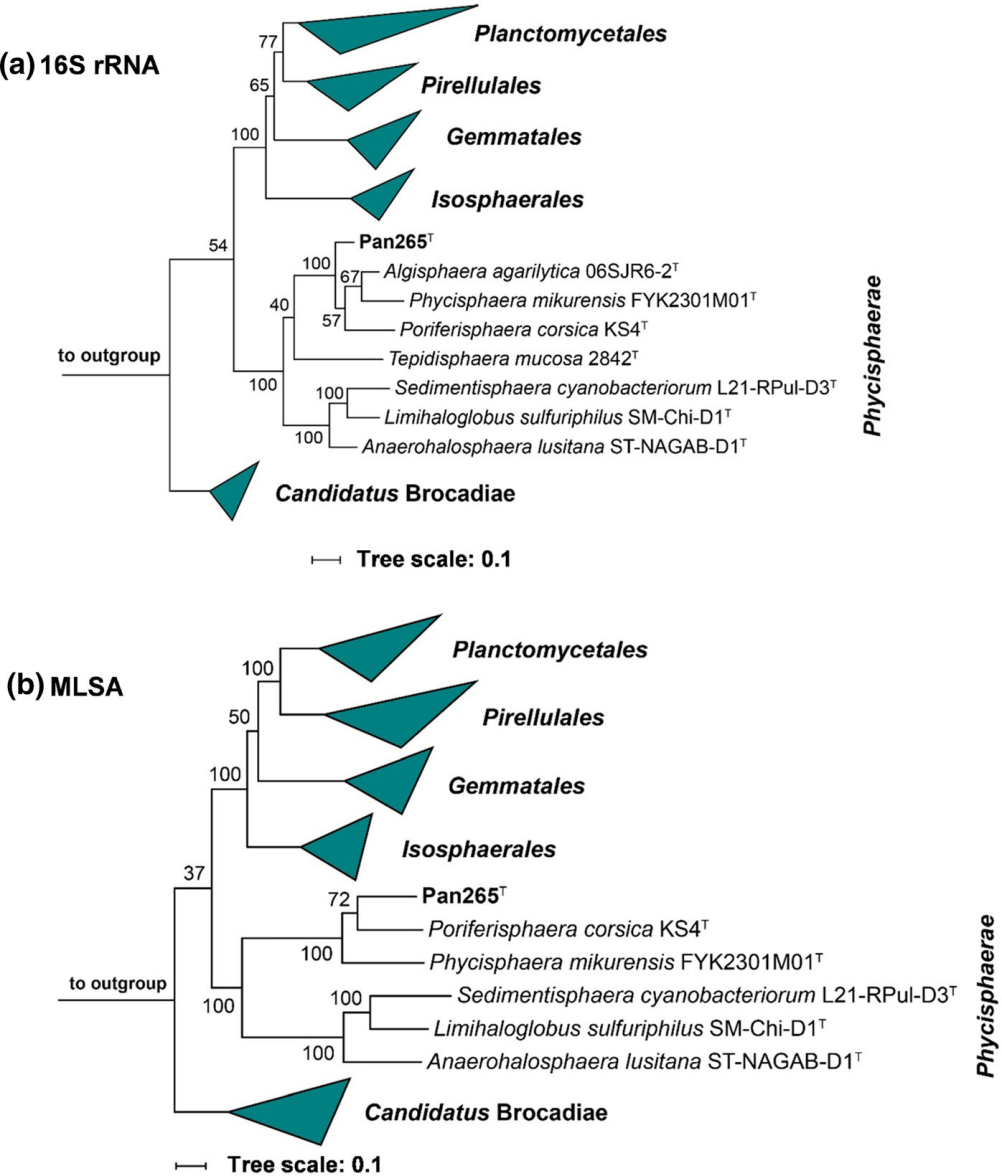
Maximum likelihood phylogenetic analysis. 16S rRNA gene sequence- **(a)** and multilocus sequence analysis (MLSA)-based **(b)** phylogenetic trees showing the position of strain Pan265^T^. Bootstrap values after 1,000 re-samplings (16S rRNA gene) / 500 re-samplings (MLSA) are given at the nodes (in %). In the 16S rRNA gene sequence-based tree, three strains part of the PVC superphylum (outside of the phylum *Planctomycetes*), namely *Opitutus terrae* (AJ229235), *Kiritimatiella glycovorans* (NR_146840) and *Lentisphaera araneosa* (NR_027571) served as outgroup. In the MLSA-based tree, *Opitutus terrae* (CP001032.1) and *Lacunisphaera limnophila* (CP016094.1) served as outgroup

**Fig. 2:**
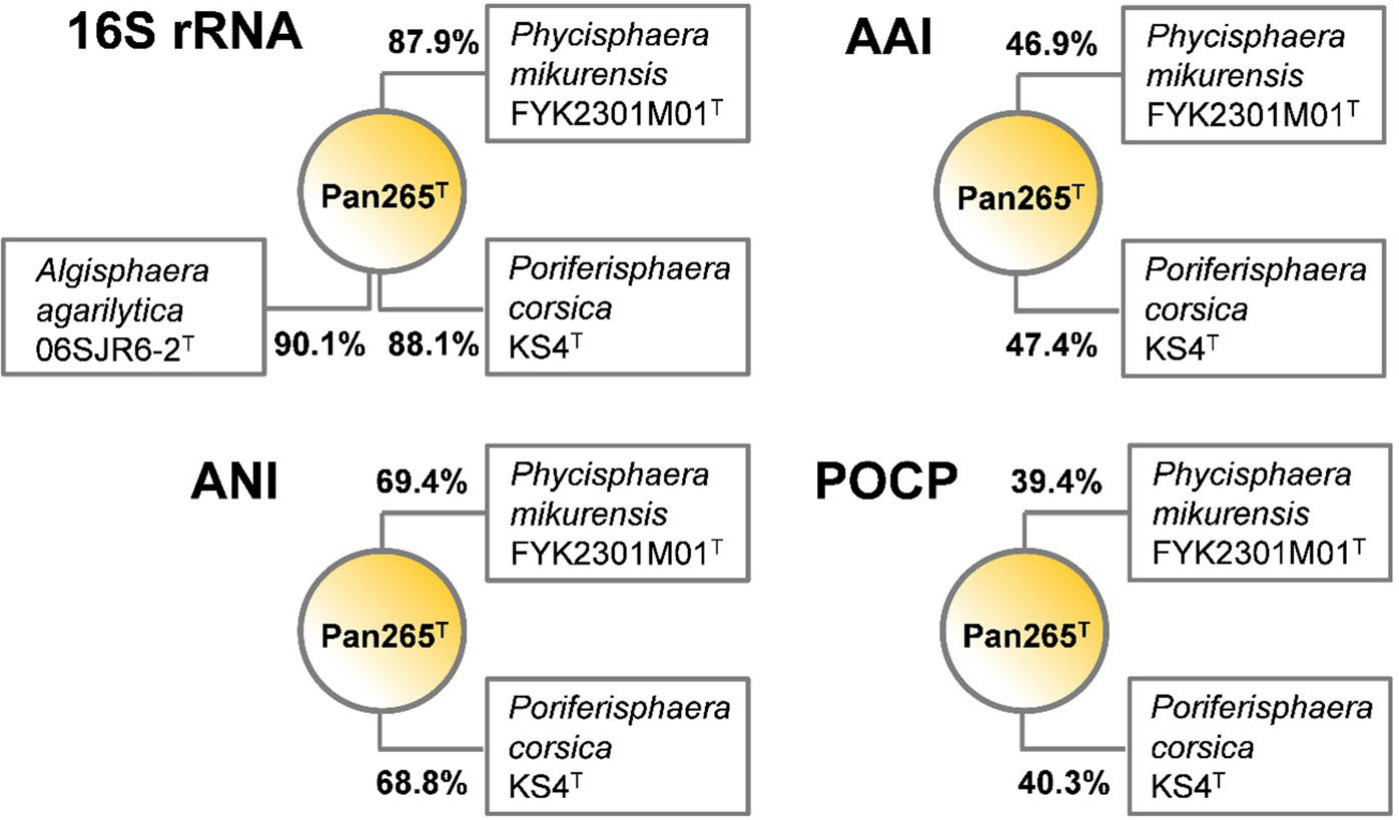
Analysis of phylogenetic markers for the delineation of strain Pan265^T^. Markers analysed: 16S rRNA gene sequence similarity (16S rRNA), average amino acid identity (AAI), average nucleotide identity (ANI) and percentage of conserved proteins (POCP). Due to the unavailability of genome sequence of *Algisphaera agarilytica* only its 16S rRNA gene sequence was used for a comparison

Strain Pan265^T^ was isolated from a red biofilm in a hydrothermal area in the Tyrrhenian Sea close to the Lipari Islands, Italy. The strain was chosen for subsequent analyses after it was identified as a member of the phylum *Planctomycetes* based on its 16S rRNA gene sequence. To determine the phylogenetic position of strain Pan265^T^ in the phylum *Planctomycetes* based on the completely sequenced genome, we constructed maximum likelihood phylogenetic trees based on full-length 16S rRNA gene sequences and MLSA (Fig. 1) and analysed the phylogenetic markers 16S rRNA gene sequence identity, ANI, AAI and POCP using the identified closest neighbours of the novel isolate for comparison (Fig. 2). Our analysis classifies strain Pan265^T^ as a member of the class *Phycisphaerae*, more specifically of the family *Phycisphaeraceae*, which is currently the sole family in the order *Phycisphaerales.* The current closest relatives of the strain are *Algisphaera agarilytica* 06SJR6-2^ T^, *Phycisphaera mikurensis* FYK2301M01^T^ and *Poriferisphaera corsica* KS4^T^ (Fukunaga et al. 2009; Kallscheuer et al. 2020f; Yoon et al. 2014). Strain Pan265^T^ shares the highest 16S rRNA gene sequence similarity of 90.1% with *A. agarilytica*, which suggests this species as the current closest neighbour (Fig. 2). Unfortunately, the genome of this species has not yet been sequenced, so that other phylogenetic markers could not be determined for the comparison of strain Pan265^T^ to *A. agarilytica*. All obtained 16S rRNA gene sequence similarities fell below the proposed genus threshold of 94.5% (Yarza et al. 2014) (Fig. 2), indicating that strain Pan265^T^ is not a member of either of the known genera in the family *Phycisphaeraceae*. Values for POCP obtained during comparison of the novel isolate with the other two close relatives turned out to fall considerably below the proposed genus threshold of 50% (Qin et al. 2014), while AAI values of 47–48% are within the range of 45–65% for separate genera (Konstantinidis et al. 2017) (Fig. 2). ANI values of < 70% exclude that strain Pan265^T^ belongs to any already described species (species threshold 95%) (Kim et al. 2014). Taking all available information into consideration, there are no arguments speaking against assignment of strain Pan265^T^ to a novel genus; in case that this impression is also supported by sufficient differences from comparison of phenotypic and genomic characteristics.

### Morphological and physiological analyses

**Fig. 3:**
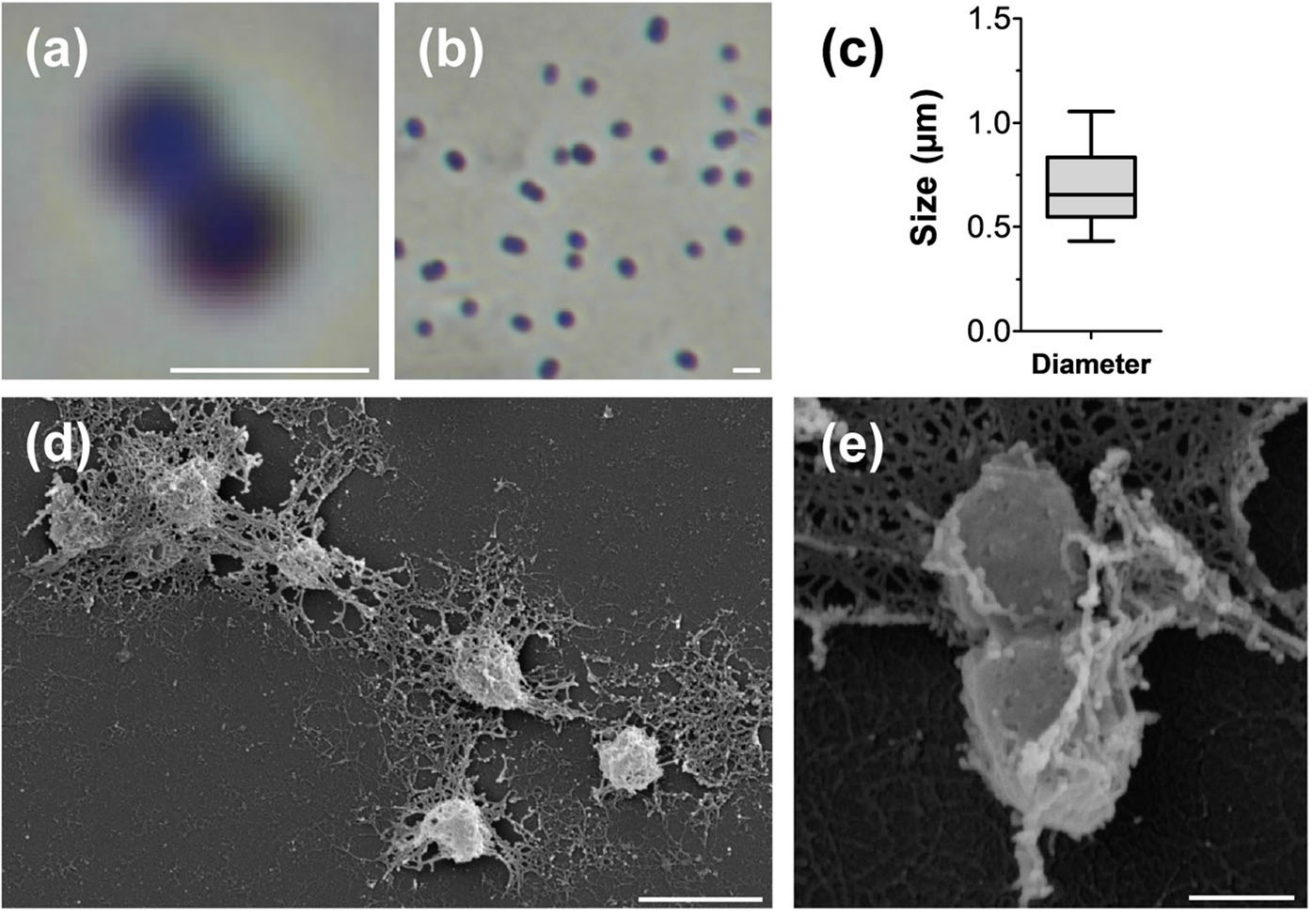
Microscopy images and cell size plot of strain Pan265^T^. The mode of cell division **(a)** and a general overview of cell morphology **(b, d, e)** is shown in the micrographs. For determination of the cell size (**c**) at least 100 representative cells were counted manually or by using a semi-automated object count tool. The scale bars represent 1 µm

The analysis of morphological and physiological characteristics of strain Pan265^T^ was performed using exponentially growing cells harvested from M1H NAG ASW medium. The results in comparison to the current closest relatives of the novel isolate are summarised in Table 1. Cells of strain Pan265^T^ are spherical, have a diameter between 0.4–1.1 µm and are motile (Fig. 3). Similar to the other described members of the family *Phycisphaeraceae*, cells lack crateriform structures, a stalk or holdfast structure and divide by binary fission. The latter is a common feature of strains within the class *Phycisphaerae*, separating its members from the class *Planctomycetia*, in which all hitherto described strains divide by budding. With regard to cell shape and size, no significant differences between strain Pan265^T^ and its close neighbours could be observed.

**Table 1:** Phenotypic and genomic features of strain Pan265^T^ compared to the close relatives *Algisphaera agarilytica* 06SJR6-2^ T^, *Poriferisphaera corsica* KS4^T^ and *Phycisphaera mikurensis* FYK2301M01^T^. The genome analysis is based on GenBank accession numbers CP036280.1 (Pan265^T^), CP036425.1 (*P. corsica* KS4^T^) and NC_017080.1 (*P. mikurensis* FYK2301M01^T^). The genome of *Algisphaera agarilytica* 06SJR6-2^ T^ has not yet been sequenced. n.o. not observed, n.d. not determined

Feature	Pan265^T^	*A. agarilytica* 06SJR6-2^ T^	*P. corsica* KS4^T^	*P. mikurensis* FYK2301M01^T^
*Phenotypic features*				
Shape	spherical	spherical	spherical	spherical
Diameter (µm)	0.4–1.1	1.0–1.2	0.3–1.3	0.5–1.3
Colour	light pink	reddish-pink	white	pink
Temperature range (optimum) (°C)	27–36 (33)	20–30 (28)	10–33 (27)	10–30 (25–30)
pH range (optimum)	5.5–8.5 (7.0–8.0)	6.0–8.0 (7.5)	6.0–8.5 (7.5)	n.d
Division	binary fission	binary fission	inconclusive	binary fission
Motility	yes	yes	n.o.	yes
Crateriform structures	n.o.	n.d.	n.o.	no
Fimbriae	yes, polar matrix or fibre	n.d.	yes	yes
Stalk	n.o.	n.d.	n.o.	no
Holdfast structure	n.o.	n.d.	n.o.	n.d
*Genomic features*				
Genome size (bp)	3,486,541	n.d.	4,291,168	3,884,382 (3,803,225 + 81,157)
Plasmids	no	n.d.	no	1
DNA G + C (%)	63.9	63.0	48.7	73.2
Coding density (%)	91.7	n.d.	84.2	88.6
Total genes	2,913	n.d.	3,714	3,176
Genes/Mb	836	n.d.	866	817
Giant genes (> 15 kb)	1	n.d.	0	0
Protein-coding genes	2856	n.d.	3,659	3,109
Protein-coding genes/Mb	819	n.d.	853	800
Hypothetical proteins	964	n.d.	1,499	1,442
tRNAs	50	n.d.	45	59
16S rRNA genes	1	n.d.	1	1

**Fig. 4:**
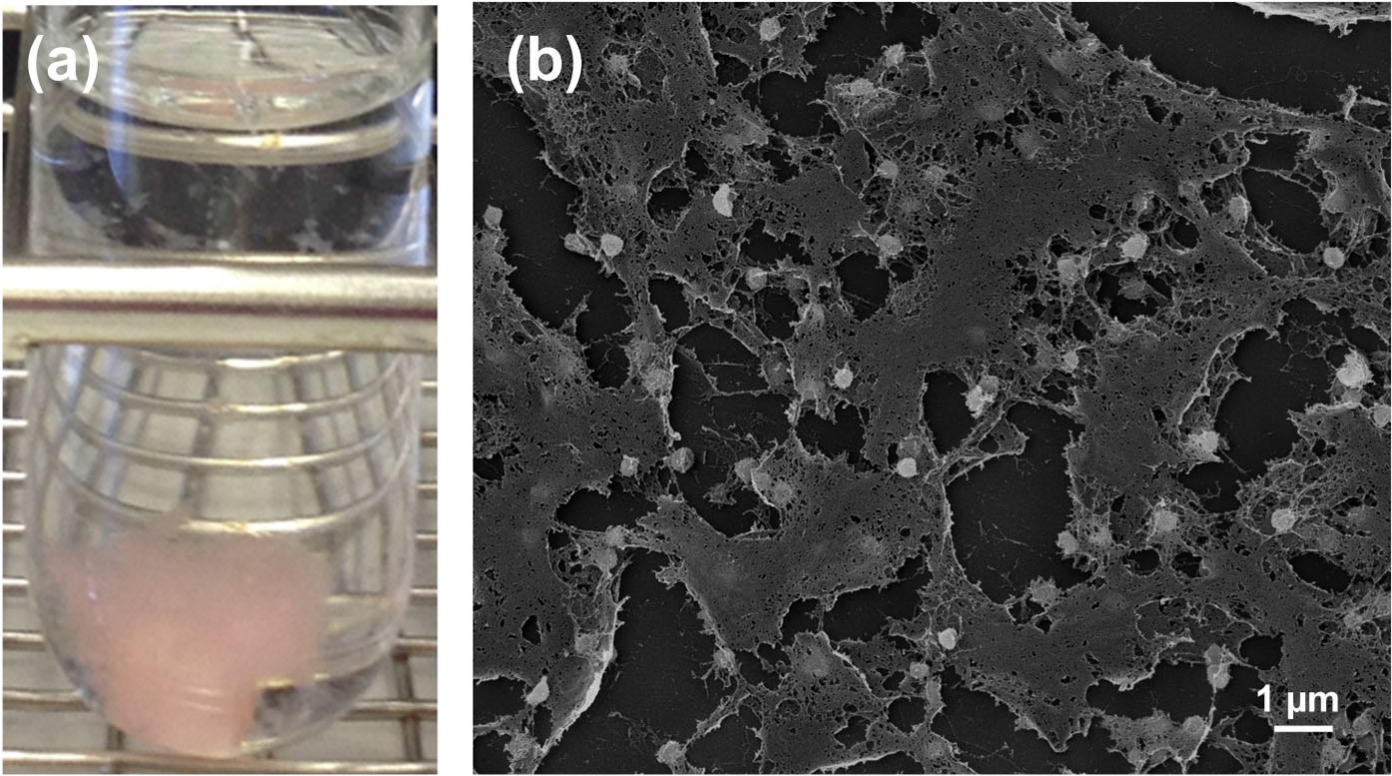
Macroscopic and microscopic visualisation of growth of strain Pan265^T^. In liquid culture, strain Pan265^T^ grows in form of a viscous colloid with a light pink pigmentation **(a)**, which likely results from formation of a dense extracellular matrix as observed during scanning electron microscopy **(b)**

**Fig. 5:**
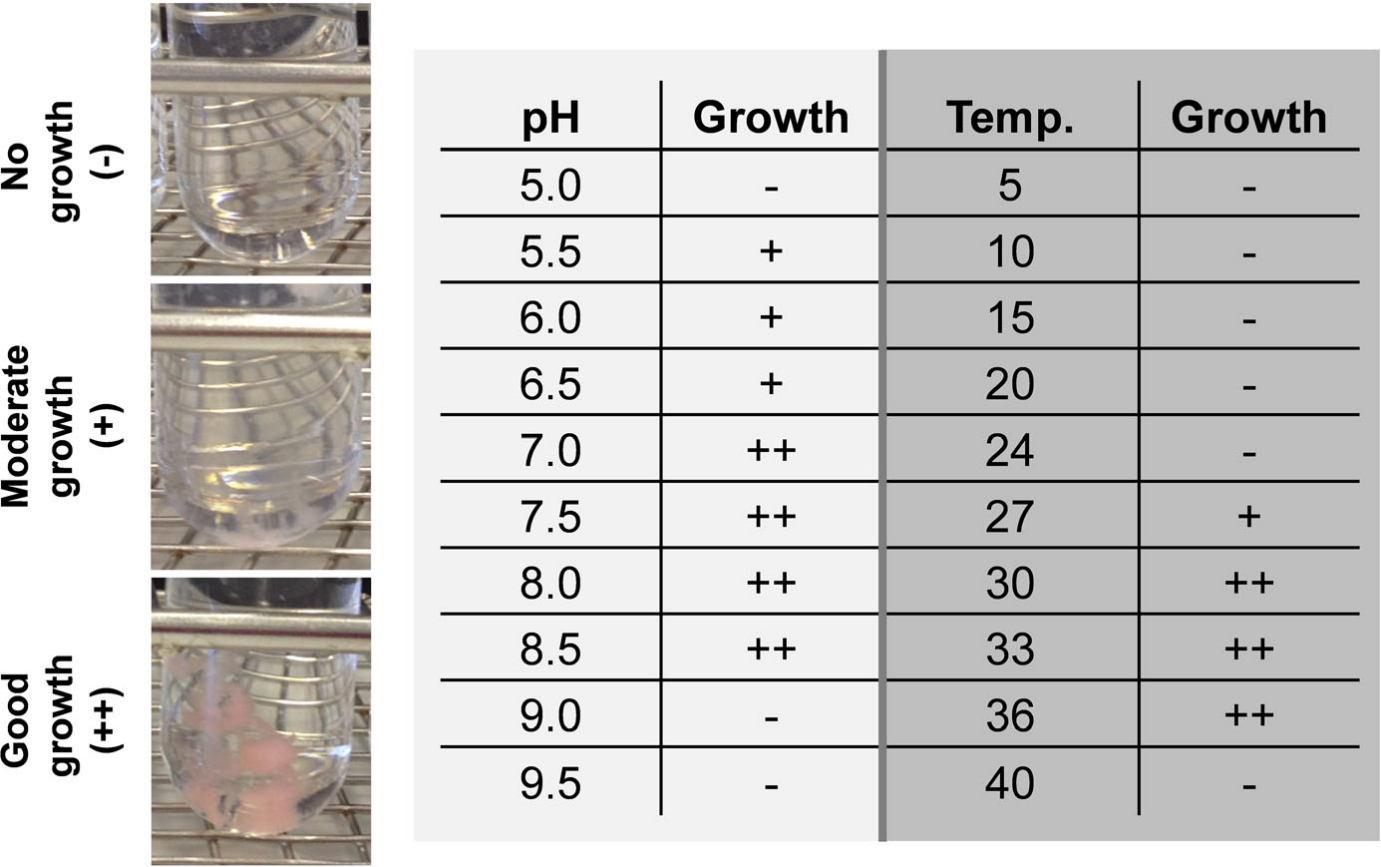
Temperature and pH optimum of strain Pan265^T^. Strain Pan265^T^ forms pink flakes during cultivation experiments in M1H NAG ASW medium for determination of temperature and pH optimum. Since determination of the cell density as OD_600_ was not possible for this strain the optimal conditions were determined by visual inspection of the tubes from cultivation experiments in biological triplicates. (-) no growth, ( +) moderate growth, (+ +) good growth

Cells of strain Pan265^T^ form a dense extracellular matrix, in which no direct cell–cell contact was observed during light and electron microscopy (except for cells in the process of division) (Figs. 3 and 4). This observation is rather uncommon given that planctomycetes typically form rosettes of 12–15 cells or larger aggregates with direct cell–cell contact. Macroscopically, the formation of an extracellular matrix by strain Pan265^T^ (Fig. 4b) leads to  growth as a viscous colloid in liquid culture (Fig. 4a). Resuspension of the gel-like aggregate was not possible, even with rigorous shaking of the culture. In consequence, photometric measurement of cell densities as OD_600_ was not possible and temperature and pH ranges allowing growth had to be determined by visual inspection of the cultivation tubes. In the cultivation experiments, strain Pan265^T^ was able to grow over a temperature range of 27–36 °C with optimal growth at 30–33 °C (Fig. 5). The strain prefers a neutral pH of 7.0–8.0, while growth was observed up to pH 5.5 and 8.5 (Fig. 5). The pH optimum of strain Pan265^T^ is comparable to its current closest relatives, while its temperature optimum is slightly higher (Table 1): Optimal growth of the three strains chosen for comparison was observed between 25 and 30 °C and all three failed to grow at 36 °C. A maximal growth rate could not be calculated for strain Pan265^T^ due to the lack of OD_600_ values.

The pigmentation of the strains within the family *Phycisphaeraceae* is quite heterogeneous, ranging from unpigmented cells to reddish-pink cells (Table 1). A light pink pigmentation was observed in case of strain Pan265^T^ (Fig. 5). Strains with such a strong difference in pigmentation despite close relationship may be interesting for future research on the role of carotenoids in planctomycetes, including the hitherto unknown metabolic pathway responsible for their biosynthesis (Kallscheuer et al. 2019).

### Genomic characteristics

When considering the range of genome sizes from 3.0–12.4 Mb for the currently described species in the phylum *Planctomycetes* (Ravin et al. 2018; Wiegand et al. 2020), members of the class *Phycisphaerae* are amongst the strains with smaller genomes. The genome of strain Pan265^T^ has a size of 3.49 Mb and is around 10% and 20% smaller than the genomes of *P. mikurensis* (3.88 Mb) and *P. corsica* (4.29 Mb), respectively. Despite the close relationship, large differences in the DNA G + C content were found (Table 1). Only *A. agarilytica* and strain Pan265^T^ have a similar DNA G + C content of 63–64%. Upon gene prediction and protein annotation with Prokka v.1.11 (Lechner et al. 2011), it turned out that the numbers of protein-coding genes per Mb (800–853) and tRNAs (45–59) are similar and all strains with available genome sequence harbour a single copy of the 16S rRNA gene. One third (33%) of the proteins of strain Pan265^T^ is of unknown function, whereas values calculated for *P. corsica* (41%) and *P. mikurensis* (47%) are considerably higher. In particular the value obtained for *P. mikurensis* is remarkable, when considering its small genome size and the expected number of essential genes required for a functional metabolism as found in canonical heterotrophic bacteria. Strain Pan265^T^ lacks plasmids, hence, *P. mikurensis* remains the only species of the current family harbouring a plasmid. In turn, strain Pan265^T^ is the only strain in the family known to harbour a giant gene coding for a single polypeptide chain longer than 5,000 amino acids (locus tag Pan265_03220, 13,493 aa).

### Genome-based analysis of carbohydrate-active enzymes and secondary metabolite-associated gene clusters

Planctomycetes are suspected to be talented degraders of high molecular weight carbohydrates derived from aquatic phototrophs as well as promising sources for novel secondary metabolites displaying interesting bioactivities. Thus, we analysed the genomes of Pan265^T^ and its close relatives for genes encoding carbohydrate-active enzymes or enzymes putatively involved in the production of secondary metabolites (Table 2). Despite the relatively small genomes of the strains Pan265^T^, *P. corsica* KS4^T^ and *P. mikurensis* FYK2301M01^T^, all three harbour between 145–175 carbohydrate-active enzymes, most of which belong to the families of glycoside hydrolases (41–52%) or glycosyltransferases (32–44%). The overall numbers as well as the distribution to the individual families for the three strains is similar.

**Table 2:** Genome-based analysis of carbohydrate-active enzymes and gene clusters potentially involved in secondary metabolite biosynthesis. The analysis is based on GenBank accession numbers CP036280.1 (Pan265^T^), CP036425.1 (*Poriferisphaera corsica* KS4^T^) and NC_017080.1 (*Phycisphaera mikurensis* FYK2301M01^T^)

Feature	Pan265^T^	*Poriferisphaera corsica* KS4^T^	*Phycisphaera mikurensis* FYK2301M01^T^
Genome size (Mb)	3.49	4.29	3.88
*Carbohydrate-active enzymes*			
Glycoside hydrolase family	85	60	79
Glycosyltransferase family	52	62	77
Polysaccharide lyase family	7	3	2
Carbohydrate esterase family	3	4	3
Auxiliary activity family	0	1	0
Carbohydrate-binding module family	15	15	14
**Total number**	**162**	**145**	**175**
*Secondary metabolite-associated clusters*			
Terpenoid	2	2	1
*N*-acyl amino acids	1	0	0
Type I PKS	0	0	0
Type II PKS	0	0	0
Type III PKS	0	0	1
NRPS	0	0	0
Type I PKS-NRPS	0	0	0
Arylpolyene	0	0	1
Bacteriocin	0	0	0
**Total number**	**3**	**2**	**3**

Numbers of gene clusters involved in secondary metabolite production typically correlate with the genome size, i.e. higher numbers are expected in strains with larger genomes. Not surprisingly, only a small number of 2–3 of such clusters was identified in the genomes of the three strains in an analysis using antiSMASH. One to two identified clusters related to terpenoid production are likely involved in the production of carotenoids in the two pigmented strains (see Table 1) but may also be relevant for steroid production (Santana-Molina et al. 2020). In addition, strain Pan265^T^ harbours a putative *N*-acyl amino acid synthase, whereas a putative type III polyketide synthase gene and a gene related to arylpolyene biosynthesis were identified in *P. mikurensis*. The three strains are only of moderate interest for the identification of novel secondary metabolites, but of major interest for elucidation of polysaccharide catabolic pathways in planctomycetes.

## Conclusion

Taken together, the novel strain shows significant differences to its current closest neighbours, especially with regard to temperature optimum, genome size and DNA G + C content. These differences support the results of the phylogenetic inference and justify the delineation of strain Pan265^T^ from the already described genera in the family *Phycisphaeraceae*. We thus propose to assign the strain to a novel species of a novel genus, for which we propose the name *Mucisphaera calidilacus*. The genus *Mucisphaera* is the fourth described genus within the family *Phycisphaeraceae*.

### Description of *Mucisphaera* gen. nov.

*Mucisphaera* (Mu.ci.sphae’ra. L. masc. n. *mucus* mucus, slime; L. fem. n. *sphaera* a ball, sphere; N.L. fem. n. *Mucisphaera* a spherical bacterium covered in slime).

Members of the genus are aerobic, mesophilic and neutrophilic heterotrophs. Cells are spherical, divide by binary fission, show motility and form polar matrix or fimbriae. The DNA G + C content is around 64%. The genus belongs to the family *Phycisphaeraceae*, order *Phycisphaerales*, class *Phycisphaerae*, phylum *Planctomycetes*. The type species of the genus is *Mucisphaera calidilacus.*

### Description of *Mucisphaera calidilacus* sp. nov.

*Mucisphaera calidilacus* (ca.li.di’la.cus L. masc. adj. *calidus* warm, hot; L. masc. n. *lacus* a lake; N.L. gen. n. *calidilacus*; of a warm lake, corresponding to the origin of the strain).

Cells are spherical (diameter 0.4–1.1 µm), lack crateriform structures, stalk and holdfast structure. Cell are embedded into an extracellular matrix without direct cell–cell contact and macroscopically appear as viscous colloid with a light pink colour in liquid medium. The type strain is Pan265^T^ (= DSM 100697^T^ = CECT 30425^T^), which was isolated from a red biofilm in a hydrothermal area in the Tyrrhenian Sea close to the Lipari Islands, Italy in September 2013. Cells of the type strain grow over a range of 27–36 °C (optimum 30–33 °C) and at pH 5.5–8.5 (optimum 7.0–8.0). The genome size of the type strain has a size of 3.49 Mb and a DNA G + C content of 63.9%

## Data Availability

The genome and 16S rRNA gene sequence are available from GenBank under the accession numbers provided in the manusript.
